# Genetic Predisposition to Severe COVID-19 Might Increase the Risk of Stroke: A Two-Sample Mendelian Randomization Study

**DOI:** 10.3389/fgene.2022.895211

**Published:** 2022-08-12

**Authors:** Jinji Zhang, Fayong Wu, Shenge Chen, Ying Zhu, Xian Luo, Xiaolin Qiu

**Affiliations:** Sanming Second Hospital, Sanming, China

**Keywords:** COVID-19, stroke, ischemic stroke, severity, Mendelian randomization

## Abstract

**Aims:** The causal relationship between COVID-19 infection and stroke has not yet been fully established. This study aimed to explore this causality using two-sample Mendelian randomization (MR).

**Materials and Methods:** Genetic variants associated with COVID-19 infection and stroke were both obtained from genome-wide association study (GWAS) summary data. The single nucleotide polymorphisms (SNPs) were selected as instrumental variables. The standard inverse variance weighted (IVW) was primarily used to assess this causality. Finally, sensitivity analysis was performed to evaluate the reliability and stability.

**Results:** The results showed that being hospitalized due to COVID-19 had a positive effect on stroke [OR = 1.05; 95% CI= (1.01, 1.10); *p* = 2.34 × 10^−5^] and ischemic stroke [OR = 1.06; 95% CI= (1.02, 1.11); *p* = 2.28 × 10^−6^] analyzed by inverse variance weighted. Moreover, the results revealed that severe respiratory symptoms due to COVID-19 had a positive effect on stroke [OR = 1.04; 95% CI= (1.00, 1.06); *p* = 0.04] and that the causal effect of severe respiratory symptoms due to COVID-19 on ischemic stroke estimated by IVW suggested a positive effect [OR = 1.06; 95% CI= (1.02, 1.09); *p* = 0.0068], too.

**Conclusion:** In summary, this study showed that severe COVID-19 might increase the risk of stroke, thus much more attention should be paid to patients with severe COVID-19.

## Introduction

Stroke, namely hemorrhagic or ischemic stroke, is a kind of acute cerebrovascular disease caused by a sudden rupture or obstruction of blood vessels in the brain, resulting in insufficient blood supply to brain tissue, and finally leading to brain damage, seriously reducing the quality of life of patients (Guzik and Bushnell 2017). Patients who suffered stroke might suffer numbness, dyskinesia, aphasia, hemiplegia, or even death (Rost 2013). According to previous studies, stroke has become a great threat to global health, with ischemic stroke, accounting for about 70% of stroke (Sacco et al., 2013; Krueger et al., 2015), having been reported to be the second leading cause of death and disability globally. In China, stroke is the primary cause of death, with the highest mortality and disability rate, and has brought a huge economic burden to society (S. Wu et al., 2019). Thus, it is important to identify every risk factor of stroke to provide guidance on the prevention and treatment in the early stage.

The advent of COVID-19 has greatly changed the ways in which healthcare operates and in which patients can access doctors. Although there are fewer patients with stroke admitted to hospital, the incidences of stroke are actually likely to be increased, as many patients fear infection from SARS-Cov-2 during hospitalization (Markus and Brainin 2020). Presently, the relationship between COVID-19 and stroke has not been fully established, as there were limited studies with regard to this relationship. Some case series, retrospective, and observational studies, with small sample sizes, showed that the incidences of stroke, especially ischemic stroke, were increased in patients who suffered from COVID-19 (Beyrouti et al., 2020; Nannoni et al., 2021; Qi et al., 2021; Trejo-Gabriel-Galán, 2020; Vogrig et al., 2021; Wang et al., 2020), in which the confounders and biases could not be eliminated, and reverse causality could not be avoided. Recently, Zuber et al. (2021) have preliminary assessed the causal relationship between COVID-19 and ischemic stroke by linkage disequilibrium score regression; the results showed that critical COVID-19 was associated with an increased risk of ischemic stroke, and multivariable Mendelian randomization analysis showed this causality was independent of other potentially mitigating risk factors. Tan et al. (2022) have also evaluated this causal relationship by Mendelian randomization study, and their results were similar to that of Zuber et al. (2021). However, both studies only focused on causal associations between critical COVID-19, which was defined as hospitalization due to COVID-19, and ischemic stroke; moreover, there were many other factors in these studies, thus, they did not assess the causal relationship between COVID-19 and stroke comprehensively. As a result, whether COVID-19 susceptibility and severity make an individual more susceptible to stroke and ischemic stroke is not clear yet. And it is of great significance to clarify the causal association between COVID-19 and stroke, which could provide guidance for the prevention and timely treatment of stroke in patients with COVID-19.

Mendelian randomization (MR) has been widely used for etiological investigations in recent years. In MR studies, the exposures are regarded as an intermediate phenotype, which are determined by genotypes, and the differences of genotypes [generally single nucleotide polymorphisms (SNPs)] are used as instrumental variables (IVs) to study the causal association between genotypes and diseases, so as to simulate the association between exposures and outcomes. Therefore, MR studies are not affected by the biases and confounders which exist in traditional epidemiological methods (such as retrospective studies), and reverse causality can also largely be avoided. Thus, the aim of this study was to assess the causal relationship between COVID-19 and stroke comprehensively using two-sample MR analysis.

## Materials and Methods

### Study Design

**FIGURE 1:**
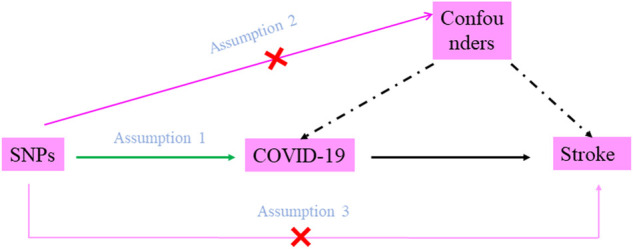
Diagram of two-sample Mendelian randomization analysis. Assumption 1: the SNPs should be associated with COVID-19 susceptibility or severity; Assumption 2: the selected SNPs should be independent of confounders; Assumption 3: the SNPs should affect stroke only *via* COVID-19 susceptibility or severity rather than via a direct correlation.

COVID-19 susceptibility and COVID-19 severity (defined as hospitalized and very severe respiratory symptoms due to COVID-19, respectively) were chosen as exposures; stroke and ischemic stroke were chosen as outcomes. There were three assumptions that should be met in the MR study: firstly, the SNPs should be closely associated with COVID-19 susceptibility or severity. In this study, significant and independent SNPs were extracted, and weak IVs were removed based on F statistics (*F* statistics = β^2^/se^2^) (*F* statistics <10) (F. Wu et al., 2020). Secondly, the selected SNPs should be independent of confounders. In this study, Phenoscanner (http://www.phenoscanner.medschl.cam.ac.uk) was used to identify and remove SNPs which were related to confounders, and the pleiotropy test was also performed. Thirdly, the SNPs should affect stroke only *via* COVID-19 susceptibility or severity rather than via a direct correlation; many MR methods were used to verify this assumption (Figure 1).

### Data Source

In this study, summary statistics for COVID-19 phenotypes were extracted from the latest version of the COVID-19 Host Genetics Initiative (HGI) GWAS meta-analyses (Round 6, June 2021) (COVID-19 Host Genetics Initiative 2020), which included genome-wide association study (GWAS) summary date with regard to different COVID-19 outcomes. In the sixth release, 2,586,691 participants of European ancestry were enrolled; there were 112,612 COVID-19 cases (with = 2,474,079 controls), 24,274 hospitalized cases due to COVID-19 (with 2,061,529 controls), and 8,779 cases who suffered from very severe respiratory symptoms due to COVID-19 (with 1,001,875 controls). Age and gender composition were unavailable in this data. All these cases and controls were of European descent. Genetic variants related to stroke and ischemic stroke were obtained from GWAS summary data published by Malik et al. (2018). There were 40,585 stroke cases and 34,217 ischemic stroke cases (with 406,111 controls) from European descent. Age and gender composition were also not available for this GWAS data.

### Instrumental Variables Extraction

The significant (*p* < 5 × 10^−8^) SNPs with minor allele frequency (MAF) > 0.01 were extracted for MR analysis, and those confounders associated with SNPs, evaluated by Phenoscanner (http://www.phenoscanner.medschl.cam.ac.uk/), were removed. When selected SNPs are in strong linkage disequilibrium (LD), biases could be introduced, so a clumping process was performed (*r* ^*2*^ < 0.5, window size = 5,000 kb) to assess whether selected SNPs were in LD.

In order to avoid reverse causality in MR analysis, we used Steiger filtering test to identify IVs that explained more of the variance in the outcome than in the exposure and removed these. Moreover, to eliminate the influence of outliers, MR-PRESSO test was used to detect and remove any potential outliers.

### Statistical Analysis

The standard inverse variance weighted (IVW) method was primarily used to analyze the causal effect of COVID-19 susceptibility and severity on stroke. Wald ratio of each SNP was calculated to assess the causal effects of each SNP on outcome, and finally the inverse variances of SNPs were used as weight for meta-analysis to evaluate the combined causal effect. The results were reported as odds ratio (OR). Furthermore, other MR methods, including MR Egger, Weighted median, Simple mode, and Weighted mode, were also used as complemental analysis. I_GX_^2^ was calculated to evaluate the violation of the no measurement error (NOME) assumption on MR Egger. *p* ≤ 0.05 was regarded as statistically significant.

### Sensitivity Analysis

Finally, sensitivity analysis was performed to assess the reliability and the stability of MR results, including heterogeneity tested by Cochran’s Q test and I^2^ statistics (I^2^ statistics>50% were regarded as significant evidence of heterogeneity.), pleiotropy tested by MR-Egger intercept and MR-RESSON test, and leave-one-out sensitivity test.

All statistical analysis were performed in R software (Version 3.6.1) with the R package “TwosampleMR.” Since published available GWAS summary data were used in this study, ethical approval was unnecessary.

## Results

### The Extraction of Single Nucleotide Polymorphisms for Mendelian Randomization Analysis

In this two-sample MR analysis, all significant (*p* < 5 × 10^−8^) and independent (*r* ^2^ < 0.5) SNPs with regard to COVID-19 susceptibility and COVID-19 severity were extracted, and weak IVs (*F* statistic < 10) were excluded. In total, eight COVID-19 susceptibility-related SNPs with a mean of *F* statistic 47.99: rs115102354, rs12482060, rs2277732, rs34326463, rs71325098, rs73062389, rs73062394, and rs75826707; 18 hospitalizations due to COVID-19-related SNPs with a mean of *F* statistic 62.95: rs115102354, rs12485445, rs13050728, rs13098271, rs143334143, rs17213,127, rs17282,391, rs2166172, rs2191031, rs2229207, rs2269899, rs2277732, rs34068335, rs35081325, rs622568, rs74956615, rs75826707, and rs7650511; and 15 severe respiratory symptoms due to COVID-19-related SNPs with a mean of 63.31: rs10860891, rs11085727, rs115102354, rs13050728, rs13089543, rs13434336, rs143334143, rs2191031, rs2237698, rs2269899, rs2277732, rs34068335, rs35081325, rs622568, and rs75826707, were retained for MR analysis. MR-RESSON test showed that there were no significant outliers. The details of these SNPs were summarized in Tables 1–3.

**TABLE 1:** The single nucleotide polymorphism selected for COVID-19 susceptibility to perform Mendelian randomization analysis.

SNP	chr	EA_exposure	EA_outcome	β_exposure	β_outcome	EAF_ exposure	EAF_outcome	se_outcome	*p*_outcome	se_exposure	*p*_exposure	*F*
rs115102354	3	G	G	0.189870	0.0323	0.06874	0.0605	0.0211	0.1260	0.032123	3.40400e-09	34.93659
rs12482060	21	G	G	0.092530	0.0029	0.33800	0.3327	0.0097	0.7605	0.016098	9.02589e-09	33.03857
rs2277732	19	A	A	0.096506	−0.0084	0.31650	0.2996	0.0108	0.4377	0.016826	9.72501e-09	32.89629
rs34326463	3	G	G	0.277170	0.0229	0.08326	0.0767	0.0181	0.2065	0.025832	7.37225e-27	115.1268
rs71325098	3	G	G	0.163760	0.0142	0.12340	0.1123	0.0153	0.3542	0.023814	6.13903e-12	47.288
rs73062389	3	A	A	0.207140	−0.0134	0.05655	0.0567	0.0257	0.6003	0.033983	1.09199e-09	37.15391
rs73062394	3	T	T	0.225990	−0.0158	0.05339	0.0481	0.0277	0.5697	0.034043	3.17103e-11	44.06794
rs75826707	3	A	A	0.290440	0.0189	0.03880	0.0267	0.0351	0.5911	0.046274	3.46099e-10	39.39479

SNP, single nucleotide polymorphism; EA, effect allele; EAF, frequency of the effect allele from the corresponding study; β, the effect of the effect allele; se, the standard error of the beta; *p*, *p*-value from the GWAS.

**TABLE 2:** The single nucleotide polymorphism selected for hospitalized due to COVID-19 to perform Mendelian randomization analysis.

SNP	chr	EA_exposure	EA_outcome	β_exposure	β_outcome	EAF_ exposure	EAF_outcome	se_outcome	*p*_outcome	se_exposure	*p*_exposure	*F*
rs115102354	3	G	G	0.44399	0.0323	0.06599	0.0605	0.0211	0.12600000	0.047412	7.64364e-21	87.69399
rs12485445	3	T	T	−0.11944	−0.0235	0.59710	0.6117	0.0100	0.01834000	0.021135	1.59302e-08	31.93707
rs13050728	21	C	C	−0.15344	−0.0006	0.64940	0.6496	0.0098	0.95350000	0.022858	1.91382e-11	45.06099
rs13098271	3	A	A	0.14366	0.0058	0.33070	0.3255	0.0102	0.56790000	0.021502	2.36810e-11	44.63895
rs143334143	6	A	A	0.22875	0.0042	0.08839	0.0866	0.0176	0.81280000	0.035580	1.28301e-10	41.33427
rs17213,127	3	T	T	0.39171	0.0488	0.04387	0.0444	0.0251	0.05210990	0.061101	1.44700e-10	41.09913
rs17282391	3	G	G	0.29118	0.0263	0.10570	0.0881	0.0171	0.12360000	0.037063	3.95185e-15	61.72228
rs2166172	1	C	C	0.12378	0.0154	0.40990	0.3758	0.0093	0.09626090	0.022275	2.74202e-08	30.87916
rs2191031	3	A	A	0.21227	0.0315	0.20510	0.1931	0.0120	0.00899705	0.030559	3.75059e-12	48.25018
rs2229207	21	C	C	0.19907	0.0221	0.08092	0.0779	0.0189	0.24330000	0.035408	1.88499e-08	31.60886
rs2269899	12	T	T	0.12406	0.0185	0.68500	0.6627	0.0096	0.05437010	0.022443	3.23802e-08	30.55637
rs2277732	19	A	A	0.17578	−0.0084	0.31440	0.2996	0.0108	0.43770000	0.023869	1.77787e-13	54.23385
rs34068335	3	T	T	0.40971	0.0142	0.12320	0.1114	0.0152	0.35090000	0.032946	1.67109e-35	154.6492
rs35081325	3	T	T	0.59312	0.0221	0.08108	0.0758	0.0182	0.22510000	0.036877	3.31665e-58	258.6866
rs622568	7	C	C	0.15423	0.0137	0.15050	0.1707	0.0123	0.26590000	0.027696	2.56502e-08	31.01013
rs74956615	19	A	A	0.27857	−0.0176	0.05013	0.0440	0.0253	0.48590000	0.049110	1.40900e-08	32.17576
rs75826707	3	A	A	0.62357	0.0189	0.03194	0.0267	0.0351	0.59110000	0.071436	2.56980e-18	76.1967
rs7650511	3	C	C	0.23715	0.0088	0.12540	0.1130	0.0223	0.69360000	0.042347	2.14102e-08	31.3618

SNP, single nucleotide polymorphism; EA, effect allele; EAF, frequency of the effect allele from the corresponding study; β, the effect of the effect allele; se, the standard error of the beta; *p*, *p*-value from the GWAS.

**TABLE 3:** The single nucleotide polymorphism selected for severe respiratory symptoms due to COVID-19 to perform Mendelian randomization analysis.

SNP	chr	EA_exposure	EA_outcome	β_exposure	β_outcome	EAF_ exposure	EAF_outcome	se_outcome	*p*_outcome	se_exposure	*p*_exposure	*F*
rs10860891	12	A	A	−0.26602	0.0098	0.89220	0.8884	0.0147	0.50320000	0.041627	1.65200e-10	40.83931
rs11085727	19	T	T	0.17929	−0.0189	0.28020	0.2756	0.0100	0.05934990	0.030197	2.89901e-09	35.25206
rs115102354	3	G	G	0.44897	0.0323	0.06100	0.0605	0.0211	0.12600000	0.058774	2.19079e-14	58.35313
rs13050728	21	C	C	−0.19735	−0.0006	0.66410	0.6496	0.0098	0.95350000	0.029635	2.74726e-11	44.34702
rs13089543	3	G	G	0.28441	0.0223	0.10410	0.0862	0.0173	0.19740000	0.045518	4.14801e-10	39.04122
rs13434336	3	C	C	0.20986	0.0110	0.30420	0.3261	0.0101	0.27670000	0.028722	2.73590e-13	53.38632
rs143334143	6	A	A	0.33972	0.0042	0.09934	0.0866	0.0176	0.81280000	0.045476	8.01124e-14	55.80559
rs2191031	3	A	A	0.30373	0.0315	0.19490	0.1931	0.0120	0.00899705	0.045643	2.84577e-11	44.28198
rs2237698	7	T	T	0.23871	−0.0223	0.08429	0.1133	0.0189	0.23910000	0.042315	1.68799e-08	31.82384
rs2269899	12	T	T	0.18841	0.0185	0.67770	0.6627	0.0096	0.05437010	0.029553	1.82600e-10	40.64478
rs2277732	19	A	A	0.24312	−0.0084	0.32210	0.2996	0.0108	0.43770000	0.029940	4.65050e-16	65.93831
rs34068335	3	T	T	0.47823	0.0142	0.11420	0.1114	0.0152	0.35090000	0.041968	4.42690e-30	129.8485
rs35081325	3	T	T	0.68803	0.0221	0.07389	0.0758	0.0182	0.22510000	0.046813	6.69576e-49	216.014
rs622568	7	C	C	0.22722	0.0137	0.13910	0.1707	0.0123	0.26590000	0.037705	1.67900e-09	36.31577
rs75826707	3	A	A	0.67025	0.0189	0.03298	0.0267	0.0351	0.59110000	0.088230	3.03879e-14	57.70867

SNP, single nucleotide polymorphism; EA, effect allele; EAF, frequency of the effect allele from the corresponding study; *β*, the effect of the effect allele; se, the standard error of the beta; *p*, *p*-value from the GWAS.

### The Causal Effect of COVID-19 Susceptibility on Stroke

**FIGURE 2:**
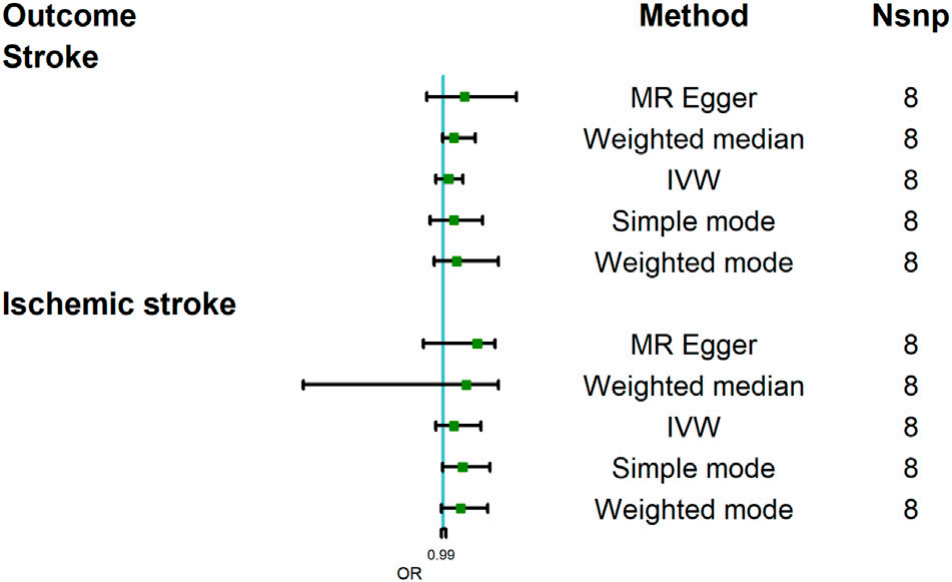
Mendelian randomization analysis results about the causal effects of COVID-19 susceptibility on stroke and ischemic stroke. CI, credibility interval.

The MR analysis results with regard to the causal relationship between COVID-19 susceptibility and stroke were summarized in Figure 2. The results showed that COVID-19 susceptibility had null effect on stroke [OR = 1.03; 95% CI= (0.96, 1.11); *p* = 0.23] analyzed by IVW, MR Egger [OR = 1.12; 95% CI= (0.91, 1.41); *p* = 0.25] and other MR methods also confirmed this result. Since weak instrumental bias could be introduced when the selected genetic variants violated the NOME assumption, we adapted I_GX_^2^ to evaluate the NOME violation on MR Egger regression. And I_GX_^2^ (90.12%) revealed the absence of NOME violation on ME Egger regression.

This study further assessed the causal relationship between COVID-19 susceptibility and stroke subtype, namely, ischemic stroke. And the result analyzed by IVW indicated that COVID-19 did not increase the risk of ischemic stroke [OR = 1.06; 95% CI= (0.96, 1.21); *p* = 0.079]; other MR methods, including MR Egger also showed a similar result [OR = 1.19; 95% CI= (0.89, 1.29); *p* = 0.28]. I_GX_^2^ (90.23%) did not indicate the existence of NOME violation on MR Egger regression. In summary, COVID-19 susceptibility did not increase the incidence of stroke.

### The Causal Effect of COVID-19 Severity-Hospitalized Due to COVID-19 on Stroke

**FIGURE 3:**
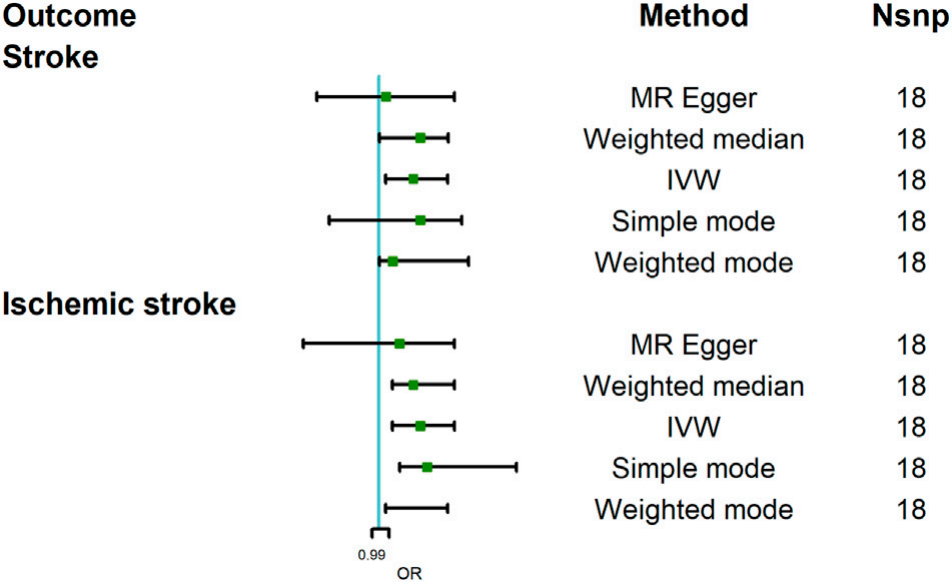
Mendelian randomization analysis results about the causal effects of COVID-19 severity (hospitalization due to COVID-19) on stroke and ischemic stroke. CI, credibility interval.

**FIGURE 4:**
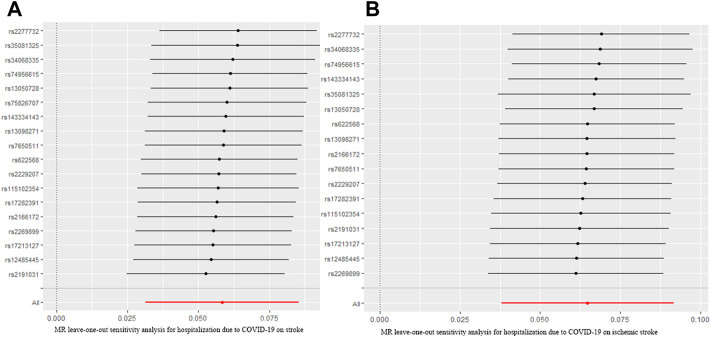
MR leave-one-out sensitivity analysis for hospitalization due to COVID-19 on stroke **(A)** and ischemic stroke **(B)**. MR, Mendelian randomization.

The MR analysis results with regard to the causal relationship between COVID-19 severity-hospitalized due to COVID-19 and stroke were summarized in Figure 3. The results showed that being hospitalized due to COVID-19 has a positive effect on stroke [OR = 1.05; 95% CI= (1.01, 1.10); *p* = 2.34 × 10^−5^] analyzed by IVW, and Weighted median results also showed a statistical significance [OR = 1.02; 95% CI= (1.001, 1.13); *p* = 0.049]. Cochran’s Q test (Q = 16.34; *p* = 0.500) and I^2^ statistics (4.01%) showed that there was no heterogeneity; MR-Egger intercept showed there was no directional pleiotropy (MR-Egger intercept = 8.29 × 10^−3^; SE = 6.36 × 10^−3^; *p* = 0.211). MR-RESSON also further confirmed the absence of pleiotropy; leave-one-out test showed that the results were stable, which were not significantly affected by any one SNP leave out (Figure 4A).

Moreover, we further assessed the causal relationship between hospitalization due to COVID-19 and ischemic stroke. Our analysis revealed that being hospitalized due to COVID-19 had a positive effect on ischemic stroke [OR = 1.06; 95% CI= (1.02, 1.11); *p* = 2.28 × 10^−6^] analyzed by IVW. Other MR methods, Weighted median [OR = 1.05; 95% CI= (1.02, 1.11); *p* = 4.11 × 10^−4^], Weighted mode [OR = 1.07; 95% CI= (1.01, 1.10); *p* = 0.009], and Simple mode [OR = 1.07; 95% CI= (1.03, 1.20); *p* = 0.018], also confirmed this causality, while MR Egger showed there was null effect of COVID-19 severity on ischemic stroke [OR = 1.03; 95% CI= (0.89, 1.11); *p* = 0.16]. However, I_GX_^2^ (86.20%) showed the presence of NOME violation (less than 90%), so inferences from this method should be interpreted with caution. Sensitivity analysis indicated that there was no heterogeneity (Q = 12.80, *p* = 0.68; I^2^ statistics = 25.02%) and directional pleiotropy (MR-Egger intercept = 4.85 × 10^−3^; SE = 6.32 × 10^−3^; *p* = 0.457), and leave-one-test showed that the results were reliable (Figure 4B).

### The Causal Effect of COVID-19 Severity-Severe Respiratory Symptoms Due to COVID-19 on Stroke

**FIGURE 5:**
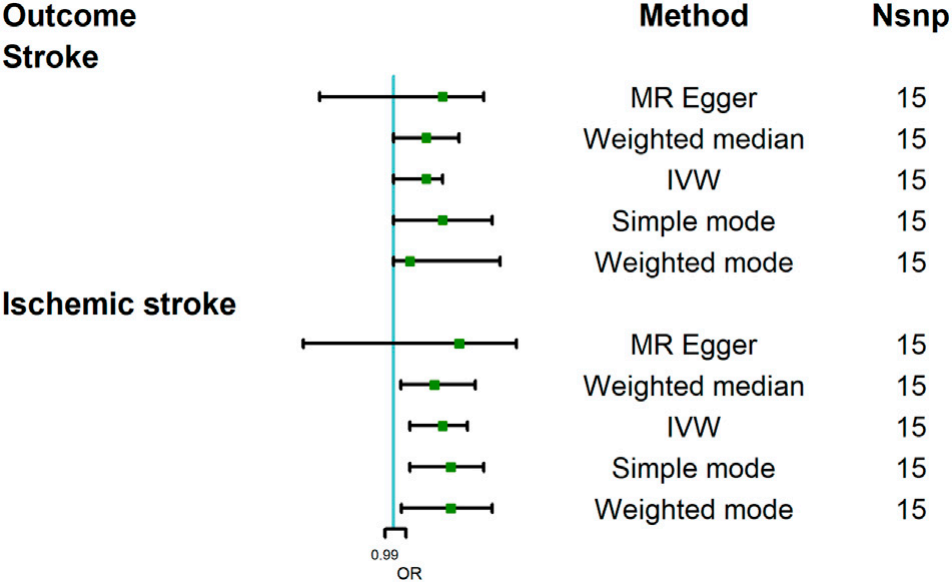
Mendelian randomization analysis results about the causal effects of COVID-19 severity (severe respiratory symptoms due to COVID-19) on stroke and ischemic stroke. CI, credibility interval.

**FIGURE 6:**
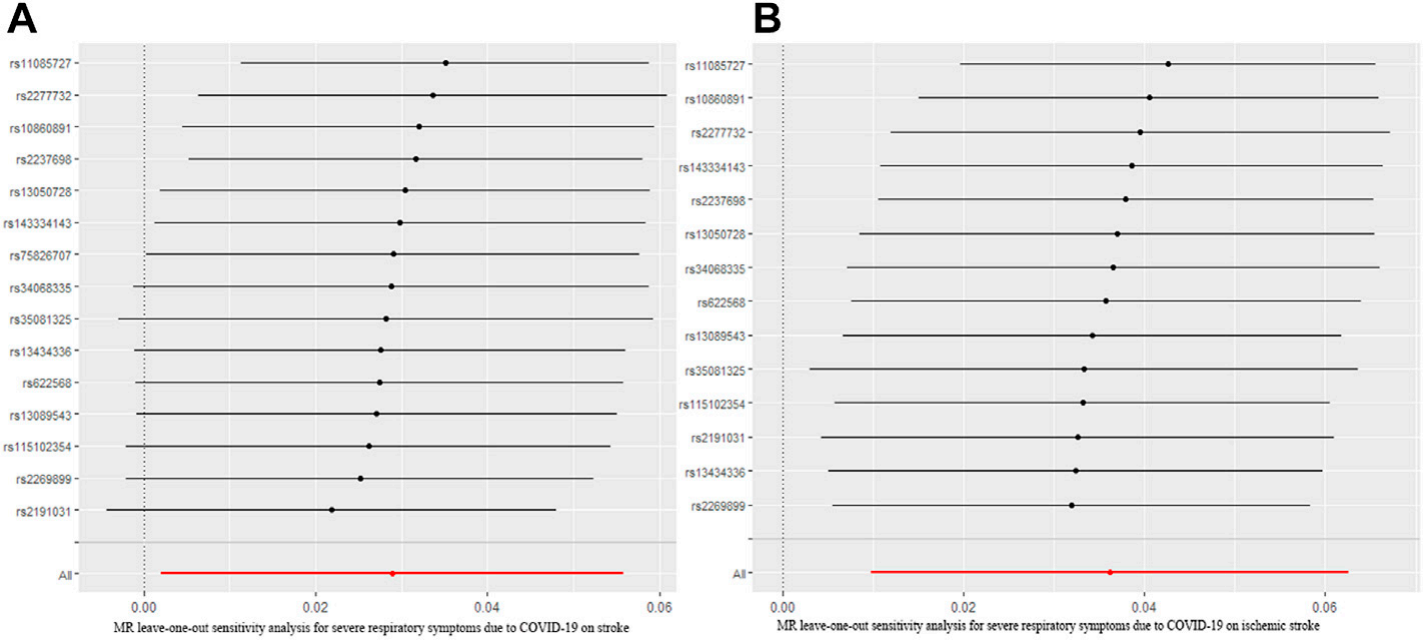
MR leave-one-out sensitivity analysis for severe respiratory symptoms due to COVID-19 on stroke **(A)** and ischemic stroke **(B)**. MR, Mendelian randomization.

To assess the causal effect of COVID-19 severity on stroke comprehensively, we further evaluated the causal association between severe respiratory symptoms due to COVID-19 and stroke. The results were summarized in Figure 5. The results revealed that severe respiratory symptoms due to COVID-19 had a significant positive effect on the risk of stroke [OR = 1.04; 95% CI= (1.00, 1.06); *p* = 0.04], which was analyzed by IVW. There was no heterogeneity based on Cochran’s Q test (Q = 19.54; *p* = 0.15) and I^2^ statistics (28.36%); MR-Egger intercept test (MR-Egger intercept = −0.008; SE = 0.01; *p* = 0.435) and MR-RESSON indicated the absence of directional pleiotropy. And these results were stable based on leave-one-out test (Figure 6A).

The causal effect of severe respiratory symptoms due to COVID-19 on ischemic stroke estimated by IVW also suggested a positive effect [OR = 1.06; 95% CI= (1.02, 1.09); *p* = 0.0068], and this causality was also further confirmed by Weighted median [OR = 1.05; 95%CI= (1.009, 1.10); *p* = 0.005], Weighted mode [OR = 1.07; 95% CI= (1.01, 1.12); *p* = 0.041], and simple mode [OR = 1.07; 95% CI= (1.02, 1.11); *p* = 0.046], while MR Egger showed null effect, however, I_GX_^2^ (80.11%) indicated the existence of NOME violation (less than 90%) on MR Egger, so this result was not adapted. The sensitivity analysis suggested that there was no heterogeneity (Q = 18.12, *p* = 0.15; I^2^ statistics = 28.26%) or directional pleiotropy (MR-Egger intercept = −7.07 × 10^−3^; SE = 0.010; *p* = 0.49), and leave-one-out test indicated that the results were reliable and stable (Figure 6B).

## Discussion

COVID-19 has infected millions of people worldwide since 2019, causing a huge challenge for global health. Although acute respiratory symptoms are the primary clinical manifestations for COVID-19, many other symptoms could also be present, and multiple organs’ function could be impaired due to cytokine storm or cytokine release syndrome caused by COVID-19. Stroke was reported to be one of the main complications of COVID-19 (Qi, Keith, and Huang 2021). Many cases series and retrospective studies have showed that COVID-19 is a risk factor for stroke (Beyrouti et al., 2020; Nannoni et al., 2021). A system review published by Montalvan et al. (2020) revealed that about 48.8% of COVID-19 patients suffered from cerebrovascular diseases, in which ischemic stroke accounts for 87.5%. Another meta-analysis performed by Nannoni et al. (2021) reported that the incidence of acute cerebrovascular diseases was about 1.4% in patients with COVID-19, and ischemic stroke was the most common type of cerebrovascular diseases, about 87.4%. Recently, Siepmann et al. (2021) performed an observational study, and, from comparing patients with severe COVID-19 to patients without, drew the conclusion that the risk of acute stroke was increased in patients with severe COVID-19 [RR = 4.18, 95% CI = (1.70, 10.25); *p* = 0.002]. It seemed that COVID-19 was a risk factors for stroke, especially ischemic stroke. However, most of above studies were cases series, retrospective, and observational studies, thus the causal relationship between COVID-19 or severe COVID-19 and stroke or ischemic stroke was not yet clear.

As a novel strategy for causality research, MR appealed to investigators and had been widely applied for various studies in recent years (Aung et al., 2020; Ponsford et al., 2020; Au Yeung et al., 2021). In the present work, we used two-sample MR to explore the causal relationship between COVID-19 and stroke. On the one hand, our results showed that COVID-19 susceptibility had null effect on stroke and ischemic stroke estimated by IVW. On the other hand, we revealed that severe COVID-19 might increase the risk of stroke and ischemic stroke. Specifically, our results suggested that being hospitalized due to COVID-19 had positive causal effects on stroke and ischemic stroke. Moreover, it was also proved that severe respiratory symptoms due to COVID-19 had a positive effect on stroke and ischemic stroke; all these results were reliable and stable, tested by sensitivity analysis. In summary, these results showed that severe COVID-19 might increase the risk of stroke, especially ischemic stroke.

The specific mechanisms of stroke in patients with COVID-19 are not fully clear. According to previous articles (Spence et al., 2020; Wang et al., 2020), the plausible mechanisms of hemorrhagic strokes were the increased affinity of SARS-COV-2 to angiotensin-converting enzyme 2 (ACE2) receptors, which were mainly expressed in endothelial and arterial smooth muscle in brain tissues, allowing SARS-COV-2 to invade and cause damages to cerebral blood vessels, and finally leading to blood vessels rupturing. As for ischemic stroke, the specific mechanisms are not yet clear, and might be multifactorial. It had been reported that 20%–55% of hospitalized patients due to COVID-19 had elevated levels of D-dimer (Wang et al., 2020), and in patients with severe COVID-19, more than 95% had increased levels of D-dimer and fibrinogen (Spence et al., 2020), indicating that hypercoagulability might be a mechanism of ischemic stroke, which was caused by inflammatory reaction and cytokine storm, leading to thrombi formation, and finally resulting in ischemic stroke. Moreover, vasculitis and cardiomyopathy also harbored a role in the pathogenesis of ischemic stroke according to previous studies (Wang et al., 2020). In total, the mechanisms of stroke in patients with COVID-19 might be multifactorial and remain to be fully elucidated.

Since our results showed that severe COVID-19 might increase the risk of stroke, much more attention should be paid to patients with COVID-19, especially with severe COVID-19, with regard to the prevention of stroke. Firstly, the epidemic has greatly changed the ways in which patients seek medical attention, thus, it is critical to ensure that patients who are unwilling to go to hospital for fear of being infected and those who live in remote areas get timely medical service. Under this circumstance, telemedicine should be widely available, from which an initial evaluation should be completed to screen stroke, and patients could get timely medical service once the symptoms appear (Markus and Brainin 2020). Secondly, for patients with COVID-19, especially with severe COVID-19, routine screenings of stroke were strongly recommended, including clinical manifestations, laboratory tests like coagulation test, imaging like brain imaging, cerebral blood vessel imaging, etc. Thirdly, for patients with severe COVID-19, secondary prevention of stroke should be carried out, and once the laboratory results showed hypercoagulability, anticoagulation therapies are recommended to prevent the occurrence of stroke.

There were some strengths to this study. Almost all previous studies with regard to the causal effect of COVID-19 on stroke were observational or retrospective studies with limited cases, in which biases and confounders could not be eliminated. Moreover, there are few studies about the potential causal relationship between severe COVID-19 and stroke. In this study, a novel method, MR analysis, was used to evaluate this potential causality comprehensively, in which confounders and biases could be minimized to a large extent, and reverse causality could also be avoided, so more precise conclusions could be drawn, and different MR methods were used to guarantee robustness. It is worth noting that there were also some limitations in this study: firstly, the present work was based on patients of a European descent, and whether this causality was applicable in other ancestries was not clear. MR analysis based on other ancestries is warranted to assess this relationship comprehensively. Secondly, in this study we focused on the effect of genetic predisposition to severe COVID-19 on stroke. Whether medical therapies for COVID-19 have a role in this relationship is unclear, so more clinical studies should be done to assess this relationship completely.

In summary, this two-sample MR analysis showed that patients with severe COVID-19 might have a higher risk of stroke, especially ischemic stroke, so more attention should be paid to the prevention of stroke in these patients.

## Data Availability

The original contributions presented in the study are included in the article/Supplementary Material, further inquiries can be directed to the corresponding author.
